# The Isolation Hospital, Southampton

**Published:** 1900-09-15

**Authors:** 


### Editor's Letter-Box.

[Our Correspondents are reminded that prolixity is a great bar to publication, and that brevity of style and conciseness of statement greatly facilitate early insertion.]

### THE ISOLATION HOSPITAL, SOUTHAMPTON.

Messrs. F. H. Greenaway and J. Arthur Smith, architects, write: In your review of new Isolation Hospital at Southampton in The Hospital of September 1st we notice a slight error which we should be glad if you will allow us to correct. You state that the main wards are heated by Doulton-Ware stoves, and from the context one would infer that no other means of heating is provided. This is not the case, as all wards throughout are heated by ventilating low-pressure hot-water radiators, and open fireplaces are provided in addition in every ward. Each of Messrs. Doulton's central stoves contains two open fireplaces.

